# Sex Doll Specifications versus Human Body Characteristics

**DOI:** 10.1007/s10508-024-02871-z

**Published:** 2024-05-16

**Authors:** Kenneth R. Hanson, Nicola Döring, Roberto Walter

**Affiliations:** 1 Department of Criminal Justice and Sociology, University of Wyoming, Laramie, WY USA; 2 Department of Economic Sciences and Media, Technische Universität Ilmenau, Ehrenbergstr. 29, 98693 Ilmenau, Germany

**Keywords:** Sex dolls, Sex robots, Sex tech, Sex toys, Racial fetishization

## Abstract

Sex dolls have been criticized for reproducing unrealistic expectations about human bodies. Yet precise sex doll measurements are lacking in the literature nor has there been any systematic attempt to determine the extent to which sex dolls exaggerate human characteristics. To address this gap, we compared the specifications of sex dolls marketed in the USA with the characteristics of women and men living in the USA. Specifically, we tested if and to what degree female dolls were slimmer (H1) and male dolls more muscular (H2) than female and male humans, respectively. Furthermore, we tested if and to what degree female dolls’ breasts (H3) and male dolls’ penises (H4) were larger than those of women and men. We also tested if sex dolls’ observed race/ethnicity was more often White than that of the US population (H5). In 2023, we collected the measures of all 757 full-body sex dolls marketed by the US retailer SexyRealSexDolls.com. Body measures from the US population were extracted from scientific literature. Descriptive and inferential statistical analyses were performed using R. All hypotheses were fully or partially confirmed, which indicated that sex dolls marketed in the USA are not realistic depictions of the US population but hypergendered (H1, H2), hypersexualized (H3, H4), and racially fetishized (H5). Implications of the lack of realism are discussed.

## Introduction

Sexual media has long been critiqued for idealizing bodies and exaggerating sexual capacities. With the development of “realistic” full-body sex dolls and sex robots, a new representation of sexualized bodies has arrived. Sex dolls are a type of sex toy that represents full, rather than partial, bodies (Döring & Pöschl, 2018). While scholars have anecdotally commented on how sex dolls tend to be hypergendered and hypersexualized, empirical work has yet to quantify these claims (Cassidy, 2016; Hanson & Locatelli, 2022).

Widespread appearance of sex dolls and sex robots in mass media (Björkas & Larsson, 2021) and the sex tech industry’s recent boom (Hanson, 2022a; Yasir Arafat & Kar, 2021) have increased attention on sex dolls, with some scholars speculating that hypergendered and hypersexualized designs may influence people’s sexual desires (Hanson & Locatelli, 2022). The primary aim of this brief report is to empirically assess sex doll designs and compare them to human bodies so that scholars can better understand to what extent sex dolls exaggerate human characteristics. The data analyzed in this brief report cannot assess causal mechanisms between sexual desire and the use of sex dolls; however, rather than continue to speculate about sex dolls, this article presents data for scholars interested in sex doll design choices.

### Body Ideals and Sex Toys

As sex toys, the explicit purpose of sex dolls is to bring about sexual arousal and pleasure; thus, it is unsurprising sex doll advertisements highlight their sexual features (Döring, 2021). However, sex doll critics argue this technology reflects unrealistic gendered, sexual, and racial ideals that individual users may internalize (Cassidy, 2016; Richardson & Odlind, 2023). While many users are individuals, some sex dolls are used within relationships (Hanson, 2022b). Previous research shows that both individual and coupled users generally report high levels of user satisfaction (Hanson, 2022b). In addition to sexual satisfaction, individual users may benefit from a sense of companionship and using sex dolls to explore hobbies, such as erotic photography (Hanson, 2023; Hanson & Locatelli, 2022; Lievesley et al., 2023). Despite self-reported benefits, some scholars are still critical of sex dolls and sex doll users. For example, correlational evidence suggests that heterosexual males with misogynistic views are more likely to be interested in artificial companions (Desbuleux & Fuss, 2023a; Leshner & Johnson, 2024). The fear, then, is that heterosexual men’s misogynistic views of women are reinforced via the use of sex dolls. However, research also shows that sex doll users self-report a reduction in sexual activities, including risky sexual behaviors, which may suggest that sex dolls are used as sexual outlets (Desbuleux & Fuss, 2023b). Together, the empirical findings to date suggest that sex doll usage is not well understood.

Regarding their construction, some scholars argue that sex dolls are hypergendered (Cassidy, 2016). Hypergendered is defined here as how sex dolls exaggerate the secondary sex characteristics of female and male bodies (e.g., “hourglass” waist-hip ratios, muscularity). Most criticism has focused on female-sexed dolls, as they are the bulk of sex dolls currently made because the main consumer demographic so far is heterosexual men. Hanson’s (2022b) qualitative study showed that hypergendered designs can disappoint female users when models advertised as “Big and Beautiful Women” are smaller than female consumers. Although systematic study of sex dolls’ gendered construction is lacking, previous research has shown that both men and women tend to idealize masculine and feminine features and scale down less attractive body parts when making attractive digital avatars (Marković & Bulut, 2023). By extension, if sex dolls are idealized representations of attractive human bodies, it would be reasonable to expect that manufacturers might exaggerate or downplay the gendered features they believe consumers will find attractive or unattractive.

The gendered construction of sex dolls might elicit attraction, but their primary function is sexual activity. Most sex toy consumers prefer smaller, genitalia only sex toys, such as dildos, masturbation sleeves, and anal plugs (Döring et al., 2022; Rosenberger et al., 2012). Realistic-looking genitalia sex toys generally receive positive product reviews online, but many of the most popular models are abstract in both form and shape (e.g., pink or purple cylinders rather than flesh-toned replicas) (Johns & Bushnell, 2024). Without sales data, we cannot know whether realistic or exaggerated sex dolls are more popular among consumers. However, the degree to which their secondary sex characteristics resemble or exaggerate human bodies is an unanswered empirical question that can help us understand sex doll construction.

Given that many heterosexual men wished they had larger penises (Lever et al., 2006) and many heterosexual women wished they had larger breasts (Forbes & Frederick, 2008), it is possible sex dolls are built to embody these desires as well. For this study, we refer to exaggerated breasts and penises as hypersexualized rather than hypergendered to differentiate between features that are explicitly advertised for their sexual appeal as sex toys.

Little research on how sex dolls embody racialized characteristics, or how sex doll racial characteristics appeals to users’ desires, exists. It is possible that there is a preference for White sex dolls. Current empirical research finds that many sex doll owners are White and live in Western countries (Hanson & Locatelli, 2022) and that a primary motivation for purchasing sex dolls is the ability to easily meet all of one’s emotional and sexual desires (Hanson, 2022b; Lievesley et al., 2023). Furthermore, as the durability of racial tensions and other structural factors have contributed to racial homogamy despite globalization and immigration, it is possible users would purchase a sex doll of their own race (Jacobson & Heaton, 2008). Alternatively, it is possible that sex doll usage overlaps with racial fetishes because as a non-human partner, sex doll users might feel safer exploring their desire for different racial and ethnic partners. If the latter is the case, we might expect Black and Asian dolls to be of particular interest given the popularity of Black and Asian pornography among White Western porn consumers (Miller-Young, 2014; Zhou & Paul, 2016).

### State of Research

Although no study has compared sex dolls to human bodies, previous research has sought to understand whether human replicas are proportional analogs. Most famously, Norton et al. (1996) found Barbie and Ken dolls do not scale up to human dimensions. Specifically, Barbie dolls are less proportional to human females than Ken dolls are to human males. Although Barbie and Ken dolls are not explicitly sexual, albeit obviously gendered, it might be possible to understand sex dolls as both physical representations and sexual media.

Some have posited that sex dolls are pornographic sexual media that could influence people’s desires and/or self-worth (Richardson & Odlind, 2023). The causal mechanisms of pornography’s impact on people’s desires and self-worth are not well understood. For example, Maheux et al.’s (2021) study of US girls aged 15–18 found higher levels of pornography consumption were positively correlated with self-objectification and body comparison. However, pornography consumption did not positively correlate with body shame, suggesting a complicated relationship between people’s consumption of pornography and sense of self-worth. This finding mirrors Vaillancourt-Morel et al.’s (2019) review of pornography scholarship which found that context is an important moderator. Analyzing sex dolls as objects for personal use may thus fail to account for what needs they meet and how they are actually used. As Döring and Pöschl (2020) found, sex toy use in both partnered sex and solo sex was viewed favorably by women and men, suggesting that, in certain contexts, the use of sex dolls may be desirable. Indeed, small-scale qualitative studies have found partnered sex doll users (Hanson, 2022b). Sex toys use is also viewed favorably by sexual minorities (Rosenberg et al., 2012), and the flexibility of sex doll customizability has been shown to be of interest to sexual minority users (Hanson, 2022b). In sum, while understanding how sex dolls physically represent human bodies is important, the benefits and consequences of their designs must be contextually interpreted.

### Current Study

This study analyzed sex doll specifications because they are a relatively new embodied artifact designed for sexual activity. Little empirical research on sex dolls designs exists (Döring & Pöschl, 2018; Döring et al., 2020; Hanson & Locatelli, 2022; Lievesley et al., 2023). By examining sex dolls’ specifications, we can better gauge how they may or may not realistically represent human bodies.

Based on previous research on idealized gendered representations, the purpose of sex toys, and current ideas about gender, race, and sexual attractiveness outlined above, we offer the following research hypotheses.

We hypothesize sex dolls are hypergendered by fulfilling the feminine body ideal of slimness and the masculine body ideal of muscularity to a significantly higher degree than humans:

#### H1

Female sex dolls have slimmer bodies than female humans in the USA.

#### H2

Male sex dolls have more muscular bodies than male humans in the USA.

We hypothesize sex dolls are hypersexualized by exaggerating female breast size and male penis size:

#### H3

Female sex dolls have larger breasts than female humans in the USA.

#### H4

Male sex dolls have larger penises than male humans in the USA.

Finally, given most sex doll users in Western countries are White, we hypothesize sex dolls are predominantly White:

#### H5

Both female and male sex dolls are disproportionally White compared to female and male humans in the USA.

## Method

This study was based on quantitative content analysis of sex doll descriptions and studies of human bodies. This approach extends previous work analyzing visual media and cultural artifacts (Harriger et al., 2023; Martins et al., 2011; Quinn et al., 2022). To ensure transparency and reproducibility, the codebook, data file, and analysis script for R are available (https://osf.io/ym9d2/).

### Sampling

#### Sample of Sex Doll Specifications

The US third-party doll retailer *SexyRealSexDolls* (https://sexyrealsexdolls.com/) was sampled because they are among the largest doll retailers and display their products with detailed specifications. Since the website is publicly accessible, the information was analyzed as publicly available content for non-commercial research.

In 2023, product specifications of 856 sex toys were manually extracted. Partial body toys (e.g., torso models; *n* = 63) were excluded because they lack full-body measurements. We excluded trans dolls because their low number did not ensure sufficient statistical power (*n* = 5). Sci-fi/fantasy dolls were excluded (e.g., elves, vampires; *n* = 31) because they were not humanlike.^1^ The final sample (*N* = 757) included 724 female-sexed and 33 male-sexed full-size humanlike dolls (see Table 1).

**Table 1:** Sample description of overall female- and male-sexed doll specifications

Overall doll body variables	Female-sexed dolls (*n* = 724)	Male-sexed dolls (*n* = 33)	Total (*N* = 757)
Price in USD *M* (*SD*)	2,681.3 (357.4)	2,893.2 (230.2)	2,690.5 (355.4)
Material	Silicone: 7.6% TPE: 92.4%	Silicone: 12.1% TPE: 87.9%	Silicone: 7.8% TPE: 92.2%
Height in cm *M* (*SD*)	161.2 (6.6)	167.9 (6.6)	161.5 (6.7)
Weight in kg *M* (*SD*)	36.8 (6.5)	44.7 (7.2)	37.1 (6.7)

Net sample comprised of humanlike full-body female-sexed and male-sexed dolls

*TPE* Thermoplastic Elastomer

Descriptive statistics (see Table 1) show sex dolls cost $2,690.5 on average, are usually TPE (Thermoplastic Elastomer), have an average height of 161.5 cm, and an average weight of 37.1 kg. Like sex differences in humans, female-sexed dolls are smaller and lighter than male-sexed dolls on average.

#### Sample of Human Body Characteristics

To compare sex doll specifications with human body characteristics, we compiled comparison measures from multiple studies as no one data set or peer-reviewed study contained every relevant measure. Inclusion criteria for studies on human body dimensions were their link to the US population and—where relevant—to younger population groups as sex dolls usually represent young adults.

For descriptive statistics of human height and weight, we used data from Wardle et al.’s (2006) study of US university students (*N* = 1,672). The description of human female and male bodies (see Table 2) shows that humans are only a few centimeters taller but nearly twice as heavy as dolls on average.

**Table 2:** Sample description of overall female and male human body characteristics

Overall human body variables	Female humans (*n* = 1,157)	Male humans (*n* = 515)	Total (*N* = 1,672)
Height in cm *M* (*SD*)	165.1 (7.4)	179.8 (8.2)	169.6 (7.6)
Weight in kg *M* (*SD*)	61.0 (9.9)	78.2 (12.4)	66.3 (10.7)

Human height and weight measures of a US university student sample taken from Wardle et al. (2006) respective sample sizes not reported in the paper were taken from Institute of Epidemiology & Health Care (2006)

#### Measures

The codebook (https://osf.io/ym9d2/) included the following measures to test the five hypotheses:

##### H1 Slimness

To assess the slimness of female-sexed dolls, we visually inspected product images and coded them (slim/not slim). Waist-to-hip ratio (WHR) (a measure of both slimness and feminine body shape) for dolls was calculated based on product information and human WHR comes from Mondragón-Ceballos et al.’s (2015) study of young female Mexican-American university students (*N* = 187). BMI was calculated using body height and body weight for dolls and human BMI measures come from Wardle et al. (2006). Obesity rates were obtained from Stierman et al.’s (2021, p. 14) study of US adults aged 20–29 (*N* = 2,489).

##### H2 Muscularity

To assess muscularity, we visually inspected images of male-sexed dolls and coded them for visible abdominal muscles (muscular/not muscular). BMI was calculated using body height and body weight for dolls and human obesity rates were based on Stierman et al. (2021).

##### H3 Breast size

Doll breast sizes were measured with advertised cup size and human breast size distribution comes from Forbes and Frederick’s (2008) study of US university students (*N* = 600). The advertised cup sizes A to D were measured as is while extra-large cup sizes (E–O) were aggregated into one group.

##### H4 Penis size

The penis lengths and circumferences of dolls were extracted from doll descriptions. Human penis measurements are based on Veale et al.’s (2014) systematic literature review of 20 studies.

##### H5: Race/ethnicity

Dolls were categorized according to the following races/ethnicities (White, Hispanic, Black, Asian, American Indian or Alaska Native, Native Hawaiian and Pacific Islander, and two or more races) based on sex doll descriptions. These racial/ethnic categories were chosen because they reflect the current US Census categories. The observed distribution was compared to a 2022 report from the US Census (*N* = 333,287,557).

#### Procedure

Using the codebook, relevant variables were manually extracted (1) from doll descriptions on the sex doll vendor website and (2) from relevant literature on human body characteristics. Quantitative measures were standardized in metric units. Measure extractions and coding were checked by all three authors to ensure reliability. The data analysis conducted with R (packages: expss, DescTools, psych, readxl, sjPlot, and tidyverse) included descriptive statistics (means, standard deviations, absolute and relative frequencies) and inferential statistics (one-sample *t* tests with Cohen’s *d* and two-dimensional chi-squared tests complemented by Cramérs V or odds ratios as effect sizes).

## Results

### Slimness and Muscularity as Indicators of Hypergendered Doll Design

Supporting H1, most female-sexed dolls were slim (82.3%) compared to 39.6% of young women in the USA (see Table 3). Furthermore, female-sexed dolls averaged a more hyper-feminine body shape than young women with a significantly lower WHR. Supporting H2, all male-sexed dolls had visible sixpacks (100.0%), while 39.9% of young men in the USA are obese (see Table 3). The BMI of female- and male-sexed dolls were both very low (< 16) due in part to their extra-lightweight design for portability. Thus, BMI may not be suitable to compare dolls and humans.

**Table 3:** Slimness and muscularity in sex dolls and humans

Body type variables	Sex dolls	Humans	Difference	Statistical test
*Slimness of female-sexed dolls*				
Slim body frequencies	*Slim body*: Yes: 82.3% No: 17.7% (*n* = 724)	*Obesity:* No: 60.4% Yes: 39.6% (*n* = 1,312)	–	*–*
Waist-to-hip ratio (WHR) *M (SD)*	0.62 (0.1) (*n* = 722)	0.77 (*n* = 187)	− 0.15	*p* < 0.001;* d* = − 2.1
Body mass index (BMI) *M (SD)*	14.2 (2.5) (*n* = 710)	22.6	− 8.4	*p* < 0.001; *d* = − 3.4
*Muscularity of male-sexed dolls*				
Muscularity: visible sixpack frequencies	*Visible sixpack:* Yes: 100.0% No: 0.0% (*n* = 33)	*Obesity:* No: 60.1% Yes: 39.9% (*n* = 1,177)	–	*–*
BMI *M (SD)*	15.7 (1.5) (*n* = 33)	24.3	− 8.6	*p* < 0.001; *d* = − 5.8

Relative frequencies, mean values (*M*), and standard deviations (*SD*). Obesity rates for age group 20–39 in the USA based on Stierman et al. (2021), *N* = 2,489. Waist-to-hip ratio based on Mondragón-Ceballos et al. (2015), *N* = 187. BMI based on Wardle et al. (2006), *N* = 1,672. Statistical tests: one-sample *t* tests. Effect sizes: *d* = Cohen’s *d*

### Breast Size and Penis Size as Indicators of Hypersexualized Doll Design

Supporting H3, female-sexed dolls’ breast size was larger than humans’ (see Table 4). About half (48.6%) of female-sexed dolls had cup sizes E–O, sizes large and infrequent enough that they are not included in many studies on young women’s breast sizes (Forbes & Frederick, 2008).

**Table 4:** Breast size in sex dolls and humans

Breast cup sizes	Female-sexed dolls	Female humans	Difference	Statistical test
A	16 (3.9%)	153 (26.2%)	− 22.3%	*p* < 0.001; *V* = 0.29
B	56 (13.5%)	224 (38.4%)	− 24.9%	*p* < 0.001; *V* = 0.27
C	62 (15.0%)	147 (25.2%)	− 10.2%	*p* < 0.001; *V* = 0.12
D	79 (19.1%)	59 (10.1%)	+ 9.0%	*p* < 0.001; *V* = 0.13
E to O	201 (48.6%)	0 (0.0%)	+ 48.6%	*p* < 0.001; *V* = 0.60
Total	414 (100.0%)	583 (100.0%)	–	*–*

Absolute and relative frequencies. Human female cup size distribution based on Forbes and Frederick (2008), *N* = 583. Statistical tests: two-dimensional chi-squared tests. Effect sizes: *V* = Cramér’s V

Supporting H4, penis sizes of male-sexed dolls were larger than humans (see Table 5): On average, doll penises were 5.6 cm longer and 2.8 cm thicker than human penises, representing large effect sizes (Cohen’s *d* > 0.8). Current data suggest a worldwide trend of increasing penile length among humans, but even the largest estimate of average erect penis length (13.93 cm) from Belladelli et al.’s (2023) study of 55,761 men globally is shorter than the average penile length of male-sexed dolls.

**Table 5:** Penis size in sex dolls and humans

Penis sizes	Male-sexed dolls	Male humans	Difference	Statistical test
Erect penis length in cm *M* (*SD*)	18.7 (4.6) *n* = 33	13.1 (1.7)	+ 5.6	*p* < 0.001; *d* = + 1.2
Erect penis circumference in cm *M* (*SD*)	14.5 (2.5) *n* = 20	11.7 (1.1)	+ 2.8	*p* < 0.001; *d* = + 1.1

Mean values (*M*) and standard deviations (*SD*). Human male penis measurements based on Veale et al. (2014), *N* = up to 15,521. Statistical tests: one-sample *t* tests. Effect sizes: *d* = Cohen’s *d*

### Whiteness as Indicator of Hyperracialized Doll Design

For H5, the overrepresentation of White sex dolls was partially supported. The underrepresentation of Hispanic/Latino/a, Black/African American, American Indian/Alaska Native, Native Hawaiian/Pacific Islander and people with two or more races was statistically significant (see Table 6). However, Asian dolls were overrepresented at 25.5% compared to 6.3% of the US population. Overall, the overrepresentation of Asian dolls and the underrepresentation of all other non-White races/ethnicities equate to both the number of White dolls and US population amounting to about 60%. However, when considering gender differences, the overrepresentation of Asian dolls is specific to female-sexed dolls. Given most dolls are female-sexed, the 26.4% of Asian female-sexed dolls biases the general estimate. Male-sexed dolls are 81.8% White.

**Table 6:** Race/ethnicity in sex dolls and humans

Race/ethnicity	Female-sexed dolls	Male-sexed dolls	Total dolls	2022 US census racial makeup estimates	Difference total dolls—humans	Statistical test
White, not Hispanic or Latino/a	59.8%	81.8%	60.8%	58.9%	+ 1.9%	*p* = 0.293; OR = 1.08
Hispanic or Latino/a	7.5%	6.1%	7.4%	19.1%	− 11.7%	*p* < 0.001; OR = 0.34
Black or African American	5.8%	6.1%	5.8%	13.6%	− 7.8%	*p* < 0.001; OR = 0.39
Asian	26.4%	6.1%	25.5%	6.3%	+ 19.2%	*p* < 0.001; OR = 5.10
American Indian or Alaska Native	–	–	–	1.3%	–	–
Native Hawaiian and Pacific Islander	–	–	–	0.3%	–	–
Two or more races	0.4%	–	0.4%	3.0%	− 2.6%	*p* < 0.001; OR = 0.13
Total	99.9%	100.1%	99.9%	102.5%	–	–

Relative frequencies. *N* = 756 dolls. *n* = 1 female-sexed Arabic doll excluded from analysis. US racial/ethnicity makeup estimates data from July 1, 2022 cover population estimates for *N* = 333,287,557 people (US Census Bureau, 2022). Totals do not equal 100% due to rounding. Statistical tests: two-dimensional chi-squared tests. Effect sizes: OR= Odds Ratios instead of Cramér’s V due to vastly different sample sizes

## Discussion

### Interpretation

Overall, the findings of this study empirically support previous research asserting that sex dolls are hypergendered and hypersexualized (Cassidy, 2016). The results show that sex dolls are only a few centimeters shorter than humans on average and mirror human sexual dimorphism with female-sexed dolls being generally smaller than male-sexed dolls, suggesting a somewhat realistic embodiment to human scale and gender differences. However, female-sexed dolls generally coupled low WHR with large breasts to create hyper-feminine designs, while male-sexed dolls generally coupled muscularity with large penises to create hyper-masculine designs. Thus, much like idealized digital humans, sex dolls accentuate feminine and masculine characteristics to make them more conventionally attractive (Marković & Bulut, 2023).

The analysis of sex dolls’ racialization presents a more complicated picture. Although White male-sexed dolls are overrepresented, Asian female-sexed dolls were also found to be overrepresented, suggesting that White sex dolls are not uniformly desired across user demographics. One interpretation of this finding is that the sample of a US retailer is catering to White men who fetishize Asian women, much like how pornographers do (Zhou & Paul, 2016). In doing so, this may reinforce stereotypes about Asian women’s docility. Another consideration is that the sample is a US retailer, but many of the manufacturers are Asian companies. At this point, little is known about non-Western markets (Hanson & Locatelli, 2022). Future research could better establish the link between Asian doll manufacturers and Western consumer markets to understand why Asian women dolls are overrepresented.

As for the overrepresentation of White male-sexed dolls, if racial fetishization was following pornographic tropes commonly found in US pornography, we might have expected to see a greater number of Black male-sexed dolls due to how Black men are hypersexualized and portrayed as being large, dominant, and having large penises (Miller-Young, 2014). Instead, we find Black male-sexed dolls are underrepresented. This may be due to the focus on men as sex doll consumers and a lack of marketing and options for women interested in sex dolls.

While the results of this study do show a certain degree of hypergendering, hypersexualizing, and racial fetishization, as previous research on pornography has demonstrated, these results must be contextually interpreted. First, certain features of sex dolls are meant to enhance their usability as a consumer product rather than depict human likeness perfectly. For example, reduced height and lower weights make sex dolls more manageable for transporting and cleaning (Döring & Pöschl, 2018). Second, like pornography, sex dolls can be used to explore sexual fantasies that a person cannot do for logistical, legal, or other reasons (Desbuleux & Fuss, 2023b). It is therefore likely that realism is only desired by a subset of sex doll users and that users have a more complicated relationship and interpretation of their sex doll use than previous research has suggested. Perhaps rather than thinking about sex dolls as exaggerating human features in ways that facilitate objectification, it is worth considering how they expand sexual capacities much in the same way that vibrators improve upon the capacity of vaginally inserted sex toys (Johns & Bushnell, 2024). For example, the flexibility of insert models may allow sex doll users to experience multiple genital configurations. In a solo sexual context, for example, genital swapping could facilitate the exploration of same-sex desires without fear of being outed. In that way, the non-humanness of sex dolls might afford users opportunities to expand their sex practices beyond those they feel comfortable enacting with a human partner.

Building upon works like Maheux et al. (2021), we suggest a need for increased literacy around sex toys, especially sex dolls and sex robots. Like the need for better porn literacy, there is a need for better sex toy literacy as scholars and nonusers alike are hasty to generalize about sex dolls and sex doll users. Sex dolls might resemble humans in some ways, but is their purpose to be exact replicas or to be objects of sexual fantasy? Sexual fantasy plays an important role in people’s sex lives, either as individuals or in relationships (Döring & Pöschl, 2020). Sex dolls might be able to be one among many sex toys or objects of pornographic consumption that can add to a person’s sex life. Nonetheless, critiques of the porn industry’s tendency to promote a limited range of sexual fantasies is relevant here, as sex dolls do clearly privilege normative ideas about bodies and desirability.

### Limitations

This study is the first to systematically compare sex doll specifications to human body characteristics. Nevertheless, some limitations exist. First, the sample from Sexyrealsexdolls.com does not include companies who exclusively sell their products on their own websites. Thus, while this sample includes 16 different brands, it is not representative of all brands. Second, by limiting the sample to human dolls and sampling from brands that only make adult dolls, this study cannot contribute to the dearth of research on the more transgressive uses of sex dolls, such as sci-fi/fantasy, animal replicas, and child-like sex dolls (Harper & Lievesley, 2022). Third, identifying and selecting studies with appropriate human body measures for comparison with sex dolls was not straightforward. Data from representative samples of the US population do not include measures on breast and penis size, and studies that include such measures rarely use representative samples. Also, some body characteristics such as race/ethnicity are age-independent while others are age-dependent (e.g., weight, WHR), hence, requiring different samples. Of particulate note, the female WHR ratio is limited to young Mexican-American women. We aimed to identify and use the most suitable reference data regarding human body dimensions that provided enough detail for statistical comparisons, but better measures collected specifically for reproducing this study would improve the reliability of our results.

### Conclusion

This study furthers our understanding of how sex dolls represent human bodies. Quantitative data analysis demonstrates that both female- and male-sexed sex dolls reaffirm normative ideals about sexual attractiveness and, within the US context, underrepresent most racial and ethnic minorities. Thus, cultural ideals about desirability seem to have been extended from sexual media to full-size human sex dolls. Future research investigating whether and to what extent cultural norms influence sex toys designs in other contexts may build upon the insights here by including other dimensions to further advance our understanding of the sex toy industry. While some of the trends analyzed in this study might be viewed as evidence of sexual objectification and/or racial fetishization, it is important to consider how material limitations, user experience, and user fantasies shape sex toy designs. In other words, while some critics may desire more “realistic” sex dolls, manufacturers have limited profitable options. We suggest a need for improved literacy when evaluating the forms and functions of sexual merchandise to better understand this emerging sexual technology.

## Data Availability

https://osf.io/ym9d2/

## Footnotes

1 Trans dolls were identified by product descriptions.

## Code Availability

https://osf.io/ym9d2/
