# Correction: Sex Doll Specifications versus Human Body Characteristics

**DOI:** 10.1007/s10508-025-03369-y

**Published:** 2025-11-25

**Authors:** Kenneth R. Hanson, Nicola Döring, Roberto Walter

**Affiliations:** 1 Department of Criminal Justice and Sociology, University of Wyoming, Laramie, WY USA; 2 Department of Economic Sciences and Media, Technische Universität Ilmenau, Ehrenbergstr. 29, 98693 Ilmenau, Germany

**Correction to: Archives of Sexual Behavior (2024) 53:2025–2033** 10.1007/s10508-024-02871-z

The following corrections have been made to the article subsequent to its original publication:

(1)

In the **Sample of Sex Doll Specifications** section, the text:

“Since the website is publicly accessible, the information is considered public domain and free to use for non-commercial purposes.”

has been corrected to read:

“Since the website is publicly accessible, the information was analyzed as publicly available content for non-commercial research.”

(2)

In the **Sample of Human Body Characteristics** section, the text:

“(*N* = 1,673)”

has been corrected to read:

“(*N* = 1,672)”

(3)

In Table 3, for male BMI, the difference value

“−12.1”

has been corrected to read

“−8.6”

(4)

In **Table 4**, the Cramér’s V values

“V = –0.29; V = –0.27; V = –0.12; V = +0.13; V = +0.60”

have been corrected by removing their nonexistent ± signs to read:

“V = 0.29; V = 0.27; V = 0.12; V = 0.13; V = 0.60”

(5)

In the **References** section, the citation:

Belladelli, F., Del Giudice, F., Glover, F., Mulloy, E., Muncey, W., Basran, S., Fallara, G., Pozzi, E., Montorsi, F., Salonia, A., & Eisenberg, M. L. (2023). Worldwide temporal trends in penile length: A systemic review and meta analysis. *The World Journal of Men’s Health, 41*, e31. 10.5534/wjmh.220203

has been corrected to read:

Belladelli, F., Del Giudice, F., Glover, F., Mulloy, E., Muncey, W., Basran, S., Fallara, G., Pozzi, E., Montorsi, F., Salonia, A., & Eisenberg, M. L. (2023). Worldwide temporal trends in penile length: A systematic review and meta-analysis. *The World Journal of Men’s Health, 41*, e31. 10.5534/wjmh.220203

